# Supplementary material for: Predicting marsh vulnerability to sea-level rise using Holocene relative sea-level data
Source: Nat Commun. 2018 Jul 12;9:2687. doi: 10.1038/s41467-018-05080-0 (PMC6043595; doi:10.1038/s41467-018-05080-0)
Supplement: Supplementary file 1 — Supplementary Information [file 41467_2018_5080_MOESM1_ESM.pdf]

Predicting marsh vulnerability to sea-level rise using Holocene relative sea-level data

Horton et al.

Supplementary information

Supplementary Figures

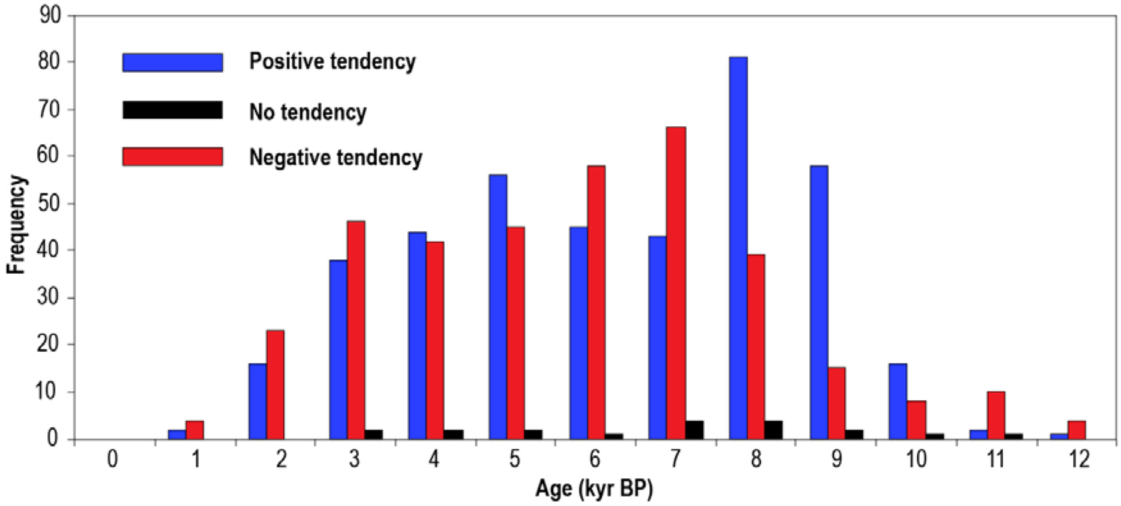

Supplementary Figure 1. Histogram of number of positive, negative and no tendency sea-level tendencies for the age of sea-level index point (1 kyr bins).

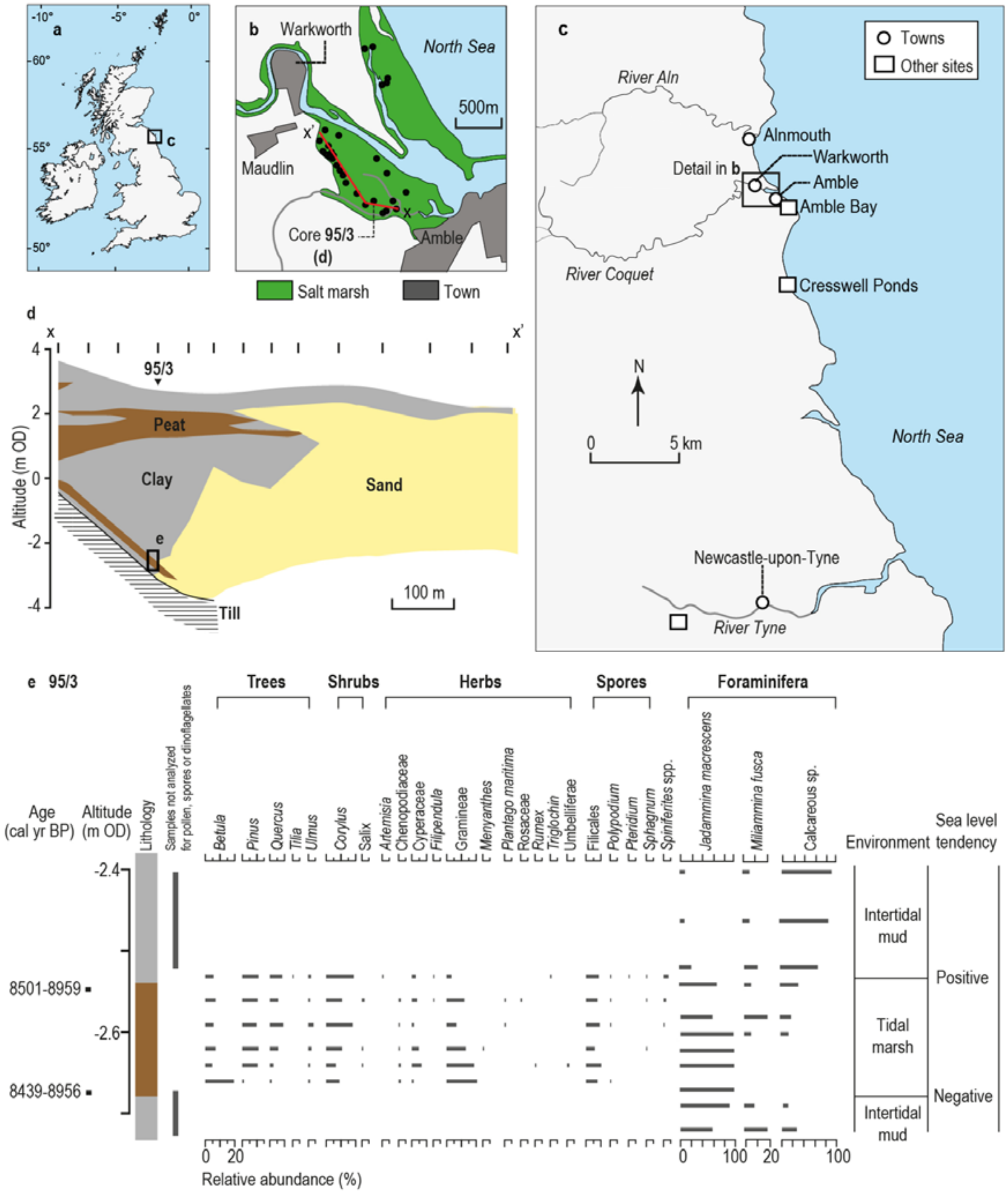

Supplementary Figure 2. Application of microfossil analyses to produce sea-level index points. (A-C) Location of Warkworth, UK. (D) Stratigraphical cross section showing position of core 95/3. OD = Ordnance datum. (E) Summary of microfossils (pollen and foraminifera) and dating results from Warkworth core 95/3 (ref. 1). Pollen frequencies are the percentage of total land pollen with a minimum count of 300 grains per level; summary foraminiferal data are actual counts. Radiocarbon dates shown as calibrated age ranges (2σ). Environmental interpretation based on sediment stratigraphy and microfossil data.

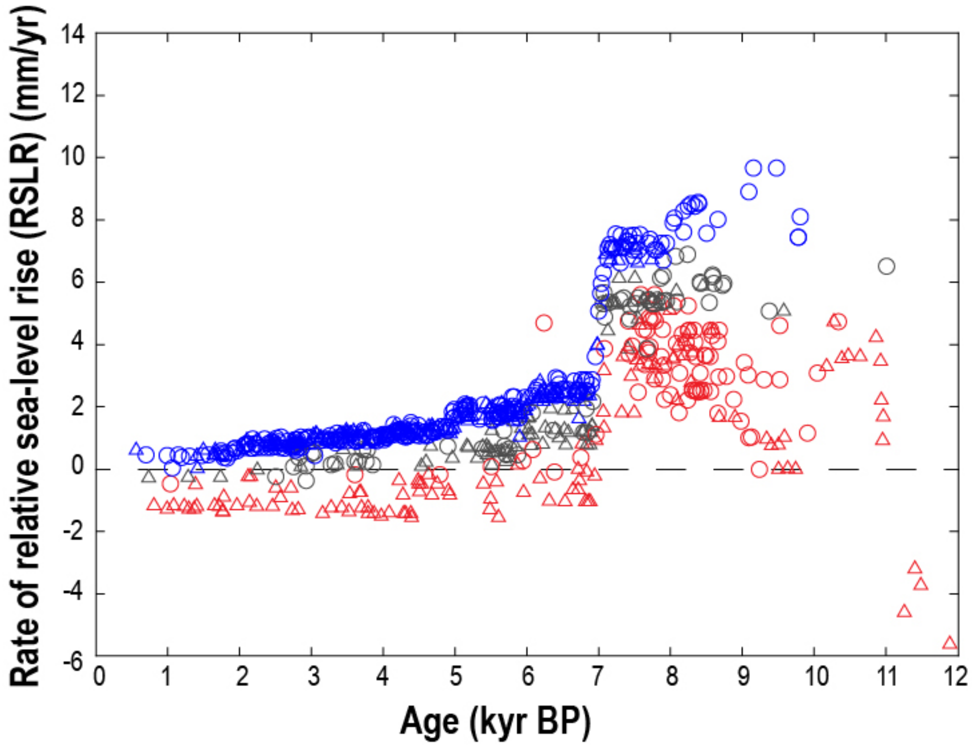

Supplementary Figure 3. Holocene rates of relative sea-level rise (RSLR) for 763 sea-level index points of the Great British database using the GIA model Bradley_71p560. Color coding reflects the separation of data into close to the center of ice loading (red), at the margin of the ice sheet (black) and sites distal from the center of ice loading (blue) (Figure 1). Circles and triangles represent data points with a positive and negative tendency respectively.

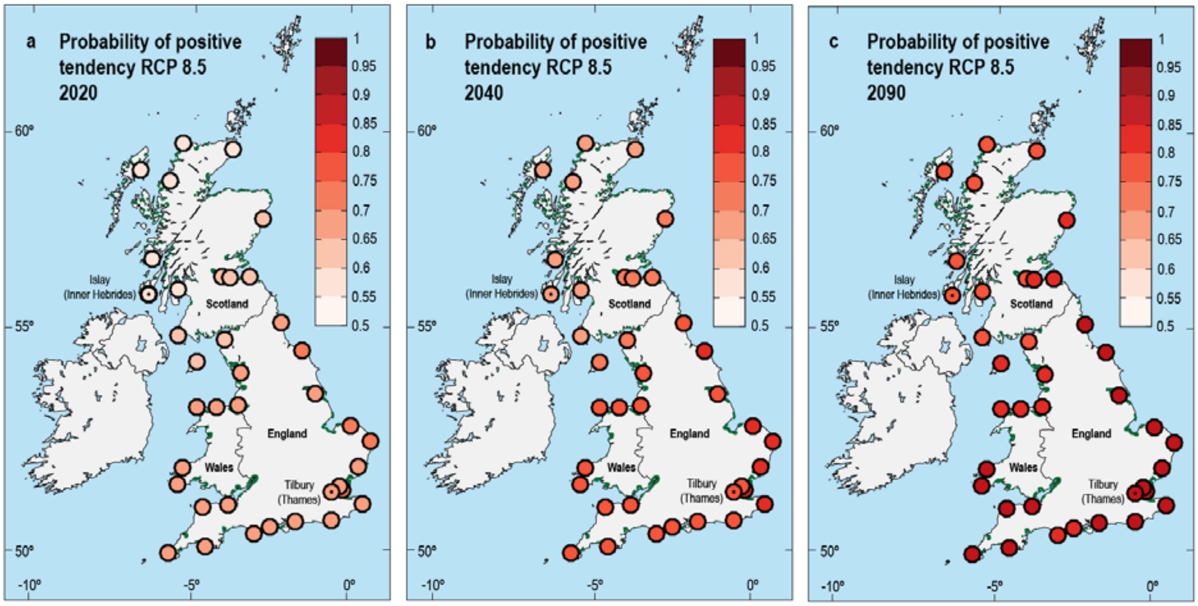

Supplementary Figure 4. Maps of selected locations in Great Britain (Supplementary Table 1) showing probability of positive sea-level tendency under high-emission RCP 8.5 pathway for (A) 2020; (B) 2040; (C) 2090. Current areas of tidal marshes (in green) following ref. 2. Tilbury and Islay are highlighted.

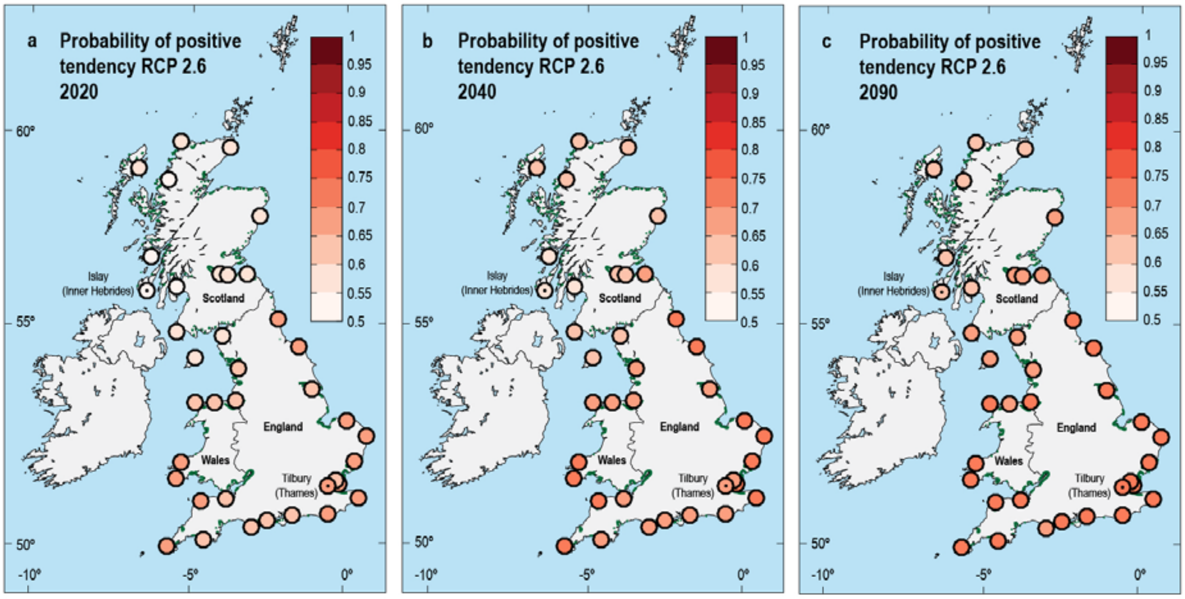

Supplementary Figure 5. Maps of selected locations in Great Britain (Supplementary Table 2) showing probability of positive sea-level tendency under low-emission RCP 2.6 pathway for (A) 2020; (B) 2040; (C) 2090. Current areas of tidal marshes (in green) following ref. 2. Tilbury and Islay are highlighted.

Supplementary Tables

Supplementary Table 1. Levels and rates of relative seal-level rise (RSLR) for tide gauge locations of Great Britain under high-emissions RCP 8.5 pathway (ref. 3). Median and (5th-95th percentile) projections shown. Tilbury and Islay are highlighted in bold.

Site	Lat	Lon	RSL rise wrt 2000 (cm)				Rate of RSL rise (mm/yr)					
			2050		2100		2010-2030		2030-2050		2080-2100	
NEWLYN	50.1	-5.54	24	(10-42)	63	(26-115)	4.5	(2.4-7.0)	6.1	(2.8-10.6)	7.9	(1.9-17.0)
DEVONPORT	50.37	-4.19	23	(9-41)	61	(24-114)	4.3	(2.1-6.8)	5.9	(2.6-10.4)	7.8	(1.6-17.0)
WEYMOUTH	50.61	-2.45	22	(7-40)	58	(21-111)	4.0	(1.8-6.6)	5.7	(2.4-10.1)	7.6	(1.4-16.8)
BOURNEMOUTH	50.71	-1.87	23	(8-41)	60	(22-113)	4.2	(2.0-6.7)	5.8	(2.5-10.3)	7.7	(1.6-16.7)
NEWHAVEN	50.78	0.06	25	(10-43)	64	(27-116)	4.5	(2.4-6.9)	6.4	(3.0-10.9)	7.9	(2.5-16.5)
PORTSMOUTH	50.8	-1.11	23	(8-41)	60	(23-113)	4.2	(2.0-6.7)	5.9	(2.6-10.4)	7.7	(1.9-16.5)
HINKLEY POINT	51.21	-3.13	23	(9-41)	60	(23-113)	4.3	(2.1-6.8)	5.9	(2.6-10.3)	7.7	(1.6-16.8)
ILFRACOMBE	51.21	-4.11	24	(9-42)	62	(25-115)	4.4	(2.3-6.9)	6.0	(2.7-10.5)	7.9	(1.7-16.9)
MILFORD HAVEN	51.71	-5.05	26	(11-43)	64	(27-117)	4.7	(2.6-7.2)	6.3	(3.0-10.7)	8.0	(1.9-17.2)
FISHGUARD II	52.01	-4.98	25	(10-43)	63	(26-116)	4.6	(2.5-7.0)	6.2	(2.9-10.6)	7.9	(1.9-16.9)
HOLYHEAD	53.31	-4.62	23	(8-41)	59	(21-112)	4.3	(2.4-6.5)	5.8	(2.2-10.6)	7.4	(1.4-16.4)
LLANDUDNO	53.33	-3.83	22	(7-41)	57	(19-111)	4.0	(2.2-6.3)	5.7	(1.9-10.6)	7.2	(1.2-16.0)
LIVERPOOL	53.4	-3	23	(8-42)	59	(21-113)	4.2	(2.3-6.4)	5.9	(2.1-10.9)	7.3	(1.6-15.9)
BIRKENHEAD	53.4	-3.02	23	(7-42)	59	(21-113)	4.2	(2.3-6.4)	5.9	(2.1-10.9)	7.3	(1.5-15.9)
HEYSHAM	54.03	-2.92	23	(6-43)	58	(19-113)	4.0	(2.2-6.2)	5.9	(1.7-11.2)	7.0	(1.5-15.5)
DOUGLAS	54.15	-4.47	19	(3-39)	50	(11-105)	3.4	(1.5-5.7)	5.0	(0.9-10.4)	6.4	(0.6-15.2)
WORKINGTON	54.65	-3.57	19	(2-39)	49	(10-104)	3.2	(1.3-5.4)	5.0	(0.8-10.4)	6.1	(0.5-14.6)
PORTPATRICK	54.84	-5.12	18	(1-37)	46	(7-101)	3.0	(1.0-5.3)	4.6	(0.4-10.0)	6.0	(0.3-14.6)
DOVER	51.11	1.32	27	(10-46)	68	(24-127)	4.7	(2.9-6.9)	6.7	(2.6-11.9)	8.4	(1.1-18.9)
SHEERNESS	51.45	0.74	28	(12-47)	69	(27-128)	4.9	(3.1-7.0)	6.9	(3.0-12.0)	8.7	(1.3-19.1)
**TILBURY**	**51.47**	**0.37**	**25**	**(10-44)**	**65**	**(23-123)**	**4.5**	**(2.6-6.7)**	**6.4**	**(2.6-11.5)**	**8.2**	**(1.2-18.3)**
SOUTHEND	51.51	0.72	25	(9-44)	64	(21-122)	4.3	(2.5-6.4)	6.3	(2.3-11.5)	8.1	(0.7-18.6)

FELIXSTOWE	51.96	1.35	26	(10-46)	67	(23-126)	4.6	(2.8-6.6)	6.6	(2.4-12.2)	8.4	(1.3-18.7)
LOWESTOFT	52.47	1.75	28	(11-48)	71	(28-130)	4.9	(3.1-7.1)	7.0	(2.8-12.4)	9.0	(1.5-19.6)
CROMER	52.93	1.3	28	(12-48)	71	(28-131)	5.0	(3.2-7.1)	7.0	(2.9-12.5)	9.0	(1.5-19.5)
IMMINGHAM	53.63	-0.19	25	(8-45)	64	(21-123)	4.3	(2.4-6.5)	6.4	(2.2-11.8)	8.0	(0.9-18.1)
WHITBY	54.49	-0.61	28	(11-49)	69	(27-128)	4.9	(2.9-7.3)	7.0	(2.8-12.3)	8.4	(1.7-18.3)
NORTH SHIELDS	55.01	-1.44	26	(9-46)	64	(22-122)	4.5	(2.4-6.9)	6.5	(2.4-11.8)	7.8	(1.2-17.4)
**ISLAY**	**55.63**	**-6.19**	**15**	**(-2-34)**	**41**	**(1-96)**	**2.4**	**(0.6-4.7)**	**4.1**	**(-0.1-9.5)**	**5.6**	**(-0.7-14.9)**
LEITH II	55.99	-3.18	19	(3-39)	50	(10-107)	3.2	(1.2-5.6)	5.1	(1.1-10.3)	6.4	(-0.2-16.0)
DUNBAR	56	-2.52	20	(4-41)	53	(12-110)	3.4	(1.4-5.8)	5.3	(1.4-10.6)	6.6	(0.0-16.3)
ROSYTH	56.02	-3.45	19	(2-39)	50	(9-106)	3.1	(1.1-5.5)	5.0	(1.0-10.2)	6.4	(-0.1-15.8)
ABERDEEN I	57.14	-2.08	20	(3-41)	52	(10-109)	3.4	(1.2-5.9)	5.3	(1.3-10.5)	6.4	(-0.2-16.0)
ABERDEEN II	57.15	-2.08	20	(3-41)	52	(10-109)	3.4	(1.2-5.9)	5.3	(1.3-10.5)	6.4	(-0.2-16.0)
WICK	58.44	-3.09	17	(-0-37)	44	(3-102)	2.8	(0.7-5.3)	4.5	(0.5-9.9)	5.7	(-1.3-15.7)
LERWICK	60.15	-1.14	17	(-0-37)	43	(1-101)	2.7	(0.5-5.2)	4.5	(0.5-9.7)	5.2	(-1.7-15.3)
MILLPORT	55.75	-4.91	16	(-1-36)	43	(4-97)	2.6	(0.8-4.8)	4.3	(0.1-9.7)	5.7	(0.0-14.4)
ULLAPOOL	57.9	-5.16	16	(-1-36)	43	(3-98)	2.6	(0.6-5.1)	4.3	(0.2-9.7)	5.7	(-0.6-15.1)
KINLOCHBERVIE	58.46	-5.05	16	(-1-37)	43	(3-99)	2.7	(0.6-5.2)	4.4	(0.3-9.8)	5.6	(-0.8-15.0)
TOBERMORY	56.62	-6.06	14	(-3-34)	40	(1-96)	2.3	(0.4-4.7)	4.0	(-0.2-9.5)	5.6	(-0.8-15.0)
STORNOWAY	58.21	-6.39	16	(-1-37)	43	(3-99)	2.7	(0.5-5.3)	4.4	(0.3-9.8)	5.8	(-0.6-15.1)

Supplementary Table 2. Levels and rates of relative seal-level rise (RSLR) for tide gauge locations of Great Britain under low-emissions RCP 2.6 pathway (ref. 3). Median and (5th-95th percentile) projections shown. Tilbury and Islay are highlighted in bold.

Site	Lat	Lon	RSL rise wrt 2000 (cm)				Rate of RSL rise (mm/yr)					
			2050		2100		2010-2030		2030-2050		2080-2100	
NEWLYN	50.1	-5.54	21	(7-38)	38	(10-84)	4.2	(2.1-6.7)	4.7	(1.2-9.4)	3.1	(-1.4-11.3)
DEVONPORT	50.37	-4.19	20	(5-37)	36	(8-81)	4.0	(1.6-6.8)	4.5	(1.1-9.1)	2.9	(-1.7-11.1)
WEYMOUTH	50.61	-2.45	18	(4-36)	33	(4-78)	3.8	(1.5-6.4)	4.2	(0.8-8.8)	2.5	(-2.1-10.7)
BOURNEMOUTH	50.71	-1.87	19	(5-36)	34	(5-79)	3.9	(1.5-6.6)	4.3	(1.0-8.8)	2.6	(-2.0-10.8)
NEWHAVEN	50.78	0.06	21	(6-39)	37	(8-83)	4.2	(1.3-7.5)	4.6	(1.7-8.7)	3.0	(-1.5-11.0)
PORTSMOUTH	50.8	-1.11	19	(5-37)	34	(5-80)	3.9	(1.3-6.9)	4.3	(1.3-8.6)	2.6	(-2.0-10.8)
HINKLEY POINT	51.21	-3.13	20	(5-37)	36	(7-81)	4.0	(1.6-6.7)	4.4	(1.1-9.0)	2.8	(-1.7-10.9)
ILFRACOMBE	51.21	-4.11	21	(6-38)	37	(9-82)	4.2	(1.9-6.8)	4.6	(1.2-9.3)	3.0	(-1.6-11.2)
MILFORD HAVEN	51.71	-5.05	22	(8-40)	40	(12-86)	4.5	(2.3-7.0)	4.9	(1.4-9.6)	3.3	(-1.3-11.6)
FISHGUARD II	52.01	-4.98	22	(7-39)	39	(11-85)	4.4	(2.2-6.9)	4.8	(1.3-9.5)	3.2	(-1.5-11.4)
HOLYHEAD	53.31	-4.62	20	(5-37)	35	(7-80)	4.0	(1.5-6.8)	4.4	(0.9-9.1)	2.9	(-1.5-10.6)
LLANDUDNO	53.33	-3.83	19	(4-37)	33	(5-78)	3.7	(1.2-6.6)	4.2	(0.6-8.9)	2.6	(-1.6-10.3)
LIVERPOOL	53.4	-3	20	(4-38)	34	(5-80)	3.9	(1.2-6.9)	4.3	(0.9-9.0)	2.7	(-1.5-10.2)
BIRKENHEAD	53.4	-3.02	20	(4-38)	34	(5-80)	3.8	(1.2-6.8)	4.3	(0.9-9.0)	2.7	(-1.5-10.2)
HEYSHAM	54.03	-2.92	19	(3-38)	33	(3-79)	3.7	(1.0-6.8)	4.2	(0.7-8.9)	2.5	(-1.6-10.0)
DOUGLAS	54.15	-4.47	16	(-0-34)	26	(-3-72)	3.1	(0.4-6.2)	3.5	(-0.1-8.3)	1.9	(-2.3-9.6)
WORKINGTON	54.65	-3.57	15	(-1-34)	25	(-5-71)	2.9	(0.2-6.0)	3.3	(-0.2-8.1)	1.7	(-2.5-9.2)
PORTPATRICK	54.84	-5.12	14	(-2-33)	22	(-7-69)	2.7	(-0.2-6.0)	3.0	(-0.3-7.7)	1.5	(-2.8-9.2)
DOVER	51.11	1.32	22	(6-42)	39	(9-86)	4.4	(1.5-7.6)	4.8	(1.6-9.2)	3.1	(-1.2-10.8)
SHEERNESS	51.45	0.74	23	(7-42)	41	(11-87)	4.5	(1.8-7.7)	5.0	(1.8-9.5)	3.3	(-1.0-10.9)
**TILBURY**	**51.47**	**0.37**	**21**	**(5-40)**	**37**	**(7-83)**	**4.2**	**(1.3-7.3)**	**4.6**	**(1.4-9.0)**	**2.9**	**(-1.4-10.5)**
SOUTHEND	51.51	0.72	20	(4-40)	35	(5-82)	4.0	(1.2-7.1)	4.4	(1.1-8.9)	2.7	(-1.6-10.3)
FELIXSTOWE	51.96	1.35	22	(6-41)	37	(7-85)	4.3	(1.4-7.4)	4.7	(1.3-9.3)	2.9	(-1.5-10.7)
LOWESTOFT	52.47	1.75	23	(7-43)	41	(10-88)	4.5	(1.6-7.9)	5.1	(2.0-9.4)	3.2	(-1.3-11.0)

CROMER	52.93	1.3	24	(8-43)	41	(11-89)	4.6	(1.6-7.9)	5.2	(2.1-9.5)	3.2	(-1.1-11.0)
IMMINGHAM	53.63	-0.19	21	(4-40)	35	(4-83)	3.9	(0.9-7.3)	4.5	(1.4-8.9)	2.6	(-1.8-10.4)
WHITBY	54.49	-0.61	24	(7-44)	41	(10-89)	4.5	(1.4-8.0)	5.2	(2.2-9.6)	3.2	(-1.2-11.0)
NORTH SHIELDS	55.01	-1.44	22	(5-42)	37	(6-85)	4.1	(1.1-7.5)	4.8	(1.5-9.3)	2.7	(-1.6-10.4)
**ISLAY**	**55.63**	**-6.19**	**11**	**(-5-30)**	**17**	**(-12-63)**	**2.2**	**(-1.0-5.9)**	**2.5**	**(-0.7-7.0)**	**1.0**	**(-3.2-8.7)**
LEITH II	55.99	-3.18	15	(-1-35)	24	(-6-72)	2.9	(-0.2-6.3)	3.5	(0.3-7.9)	1.5	(-2.9-9.3)
DUNBAR	56	-2.52	17	(0-36)	26	(-4-74)	3.1	(0.0-6.5)	3.7	(0.5-8.2)	1.7	(-2.8-9.5)
ROSYTH	56.02	-3.45	15	(-1-35)	24	(-7-71)	2.8	(-0.2-6.3)	3.4	(0.2-7.8)	1.5	(-2.9-9.3)
ABERDEEN I	57.14	-2.08	16	(-0-37)	26	(-5-74)	3.0	(-0.1-6.5)	3.7	(0.5-8.2)	1.6	(-2.8-9.5)
ABERDEEN II	57.15	-2.08	16	(-0-37)	26	(-5-74)	3.0	(-0.1-6.5)	3.7	(0.5-8.2)	1.6	(-2.8-9.5)
WICK	58.44	-3.09	13	(-3-34)	20	(-11-68)	2.4	(-0.8-6.1)	3.1	(-0.1-7.6)	1.0	(-3.5-9.0)
LERWICK	60.15	-1.14	13	(-3-33)	19	(-12-67)	2.3	(-0.8-5.9)	3.1	(-0.1-7.7)	0.8	(-3.9-9.0)
MILLPORT	55.75	-4.91	12	(-4-31)	19	(-11-65)	2.4	(-0.7-5.9)	2.7	(-0.5-7.2)	1.2	(-3.1-8.8)
ULLAPOOL	57.9	-5.16	13	(-4-32)	19	(-11-66)	2.4	(-1.0-6.2)	2.9	(-0.2-7.2)	1.0	(-3.3-8.9)
KINLOCHBERVIE	58.46	-5.05	13	(-3-33)	20	(-11-67)	2.5	(-1.0-6.2)	2.9	(-0.1-7.3)	1.0	(-3.3-8.9)
TOBERMORY	56.62	-6.06	11	(-5-30)	16	(-13-63)	2.1	(-1.3-6.0)	2.5	(-0.6-6.9)	0.8	(-3.4-8.6)
STORNOWAY	58.21	-6.39	13	(-3-33)	20	(-10-67)	2.5	(-1.2-6.7)	3.0	(0.1-7.2)	1.1	(-3.2-9.0)

Supplementary Table 3. Summary information for the location of the Great British Holocene relative sea-level database used for analysis.

Site Name	Latitude	Longitude	Region
Shetlands	60.34	-1.03	North (red)
Orkney	58.96	-2.97	North (red)
Wick	58.45	-3.12	North (red)
Dornoch Firth	57.86	-4.26	North (red)
Moray Firth	57.49	-4.46	North (red)
Coigach	58.05	-5.36	North (red)
Hebrides	57.77	-7.12	North (red)
Applecross	57.58	-5.81	North (red)
Kintail	57.28	-5.58	North (red)
Arisaig	56.93	-5.84	North (red)
Kentra	56.76	-5.84	North (red)
NE Scotland	57.66	-1.98	North (red)
Aberdeen	57.33	-1.99	North (red)
Montrose	56.70	-2.52	North (red)
Tay Valley	56.38	-3.21	North (red)
Forth Valley	56.13	-4.19	North (red)
Islay	55.81	-6.34	North (red)
Clyde	55.86	-4.49	North (red)
Ayr	55.53	-4.68	North (red)
SE Scotland	56.03	-2.69	North (red)
NE England (North)	55.69	-1.92	North (red)
NE England (Central)	55.63	-1.81	North (red)
NE England (South)	55.35	-1.60	Central (black)
NE England (Tyne)	54.96	-1.67	Central (black)
Tees	54.63	-1.23	Central (black)
N Solway Firth	55.00	-3.61	North (red)
S Solway Firth	54.90	-3.18	North (red)
Cumbria	54.39	-3.32	Central (black)
Isle of Man	54.39	-4.45	North (red)

Morecambe Bay	54.15	-2.97	Central (black)
Lancashire	53.69	-2.99	Central (black)
Mersey	53.40	-3.14	Central (black)
N Wales	53.30	-3.74	Central (black)
MWAL	52.50	-4.04	South (blue)
Humber (Inner Estuary)	53.64	-0.68	South (blue)
Humber (Outer Estuary)	53.68	-0.09	South (blue)
Lincolnshire Marshes	53.30	0.26	South (blue)
Fens	52.72	0.05	South (blue)
Norfolk	52.97	0.78	South (blue)
East Anglia	52.50	1.64	South (blue)
Bristol Channel	51.36	-2.91	South (blue)
Essex	51.63	0.62	South (blue)
Thames	51.47	0.22	South (blue)
Kent	51.04	0.97	South (blue)
Sussex	50.83	0.33	South (blue)
Hampshire	50.81	-1.35	South (blue)
SW England (Dorset)	50.65	-2.38	South (blue)
SW England (Devon)	50.42	-3.77	South (blue)
SW England (Cornwall)	50.13	-5.48	South (blue)
Kintyre	55.89	-5.46	North (red)
German Bight	55.07	6.00	South (blue)
Dogger Bank	55.02	2.98	South (blue)
Offshore (E of Yorkshire)	53.99	0.17	South (blue)
Offshore (N of Norfolk)	52.99	1.11	South (blue)
Offshore (NE of Norfolk)	53.21	2.08	South (blue)

Supplementary Table 4. Earth model parameters used in the GIA model and the associated chi squared misfit ($\chi 2$ and 95% confidence limit) calculated using the entire sea-level database of Great Britain (ref. 4, 5).

**GIA model**	**Lithosphere thickness (km)**	**Upper mantle viscosity (Pas)**	**Lower mantle Viscosity (Pas)**	**χ^2 misft (95% confidence limit)**
Bradley_71p560	71	5×10^{20}	6×10^{22}	100.39 (111)

Supplementary References

1. Shennan, I. *et al.* Holocene isostasy and relative sea-level changes on the east coast of England. *Geol. Soc. Lond. Spec. Publ.* **166,** 275–298 (2000).
2. Boorman, L. Saltmarsh Review: An overview of coastal saltmarshes, their dynamic and sensitivity characteristics for conservation and management. *JNCC Rep. 334* 132 (2003).
3. Kopp, R. E. *et al.* Probabilistic 21st and 22nd century sea-level projections at a global network of tide-gauge sites. *Earths Future* **2,** 2014EF000239 (2014).
4. Bradley, S. L., Milne, G. A., Shennan, I. & Edwards, R. An improved glacial isostatic adjustment model for the British Isles. *J. Quat. Sci.* **26,** 541–552 (2011).
5. Kuchar, J. *et al.* Evaluation of a numerical model of the British–Irish ice sheet using relative sea-level data: implications for the interpretation of trimline observations. *J. Quat. Sci.* **27,** 597–605 (2012).
